# Mind the Curve: Dose–Response Fitting Biases the Synergy Scores across Software Used for Chemotherapy Combination Studies

**DOI:** 10.3390/ijms24119705

**Published:** 2023-06-03

**Authors:** Olga Fuentealba-Manosalva, Matías Mansilla, Neudo Buelvas, Antonia Martin-Martin, Cristian G. Torres, Rodrigo A. López-Muñoz

**Affiliations:** 1 Instituto de Farmacología y Morfofisiología, Facultad de Ciencias Veterinarias, Universidad Austral de Chile, Valdivia 5110566, Chile; 2 Departamento de Ciencias Clínicas, Facultad de Ciencias Veterinarias y Pecuarias, Universidad de Chile, Santiago 8820808, Chile; 3 Laboratorio Centralizado de Investigación Veterinaria (LaCIV), Facultad de Ciencias Veterinarias y Pecuarias, Universidad de Chile, Santiago 8820808, Chile

**Keywords:** drug combinations, chemotherapy, software, synergy

## Abstract

Drug combinations are increasingly studied in the field of anticancer agents. Mathematical models, such as Loewe, Bliss, and HSA, are used to interpret drug combinations, while informatics tools help cancer researchers identify the most effective combinations. However, the different algorithms each software uses lead to results that do not always correlate. This study compared the performance of Combenefit (Ver. 2.021) and SynergyFinder (Ver. 3.6) in analyzing drug synergy by studying combinations involving non-steroidal analgesics (celecoxib and indomethacin) and antitumor drugs (carboplatin, gemcitabine, and vinorelbine) on two canine mammary tumor cell lines. The drugs were characterized, their optimal concentration–response ranges were determined, and nine concentrations of each drug were used to make combination matrices. Viability data were analyzed under the HSA, Loewe, and Bliss models. Celecoxib-based combinations showed the most consistent synergistic effect among software and reference models. Combination heatmaps revealed that Combenefit gave stronger synergy signals, while SynergyFinder produced better concentration–response fitting. When the average values of the combination matrices were compared, some combinations shifted from synergistic to antagonistic due to differences in the curve fitting. We also used a simulated dataset to normalize each software’s synergy scores, finding that Combenefit tends to increase the distance between synergistic and antagonistic combinations. We conclude that concentration–response data fitting biases the direction of the combination (synergistic or antagonistic). In contrast, the scoring from each software increases the differences among synergistic or antagonistic combinations in Combenefit when compared to SynergyFinder. We strongly recommend using multiple reference models and reporting complete data analysis for synergy claiming in combination studies.

## 1. Introduction

The use of anticancer drugs in cancer chemotherapy is often hindered by their toxicity and the high cost of new targeted therapies. Consequently, researchers have increasingly focused on studying and implementing drug repurposing and combination strategies. Drug repurposing involves the use of drugs approved for other diseases, while combination strategies entail using two or more drugs whose combined effect is greater than the individual effect of each drug [1].

In vitro studies of drug combinations have benefited from the development of several mathematical approaches. Three main principles underlying the current landscape in the field of drug combinations were first formulated in the first half of the 20th century. These principles are the Dose Equivalence Principle (DEP), which was described by Siegfried Loewe in 1926 [2], the Multiplicative Survival Principle (MSP), introduced by Chester Bliss in 1939 [3] and the Highest Single Agent (HSA), described by Sir John Gaddum in 1940 [4]. Currently, the models derived from these principles are known as the Loewe, Bliss and HSA models, respectively [5].

Each model for drug combination has its own set of assumptions and parameters. For example, the Loewe additivity model assumes that two drugs used together have an “additive” effect, which means that each drug alone produces an individual effect in in their respective dose range, and the combined effect is the sum of those individual effects. The Loewe model fits better when the two drugs have similar modes of action or target the same biochemical pathway, producing similar pharmacological effects [5]. On the other hand, the Bliss independence model postulates that the effects of two drugs are independent, and the product of their individual effects determines the combined result of the two drugs. More precisely, the Bliss model assumes a multiplicative outcome when the effect is expressed as a fraction [6]. Finally, the HSA model assumes that the effect of a drug combination equals the effect of the single drug with the highest activity. In other words, the drug with the most potent effect determines the combined effect. The HSA model is useful when one of the drugs has a much stronger effect than the other drug [5].

In addition to developing new mathematical models explaining drug combinations, since the 1990s, several informatics tools have appeared to help researchers identify the most effective drug combinations, optimize dosages, and predict drug interactions. Software programs for analyzing drug combinations provide a range of tools and models for analyzing dose–response data and determining drug synergy, antagonism, or additivity [5]. Today, many software tools are available for analyzing drug combinations, ranging from simple spreadsheet-based tools to sophisticated software packages with graphical user interfaces and machine learning algorithms. Among the most used tools, we can name MacSynergy II™ [7], CompuSyn [8], Combenefit [9], BRAID [10], SynergyFinder [11], MuSyC [12] and SiCoDEA [13].

The different software programs for drug combination analysis often employ different reference models, which can make their results not directly comparable across platforms. For example, Compusyn and MacSynergy II™ are based on the Loewe additivity model [7,14], while BRIAD and MuSyC were developed using new mathematical models that aim to incorporate the three fundamental principles of drug synergism [10,12]. Additionally, some software, such as Combenefit or SynergyFinder, analyze data using multiple models including Loewe, Bliss, and HSA, offering a range of tools and models for analyzing dose–response data and assessing drug synergy [9,11].

Compusyn and SynergyFinder are widely used software programs for drug combination analysis. However, a more comprehensive evaluation of their features is necessary. Meyer et al. (2020) conducted an extensive review, comparing both software using a large dataset of antimalaria drugs. The study revealed conflicting results between the synergy/antagonism scores generated by both programs [15]. Despite this, further investigation is required to fully understand the underlying causes of these discrepancies.

This study compares the performance of Combenefit and SynergyFinder for analyzing drug combinations involving two non-steroidal analgesics (NSAIDs), celecoxib and indomethacin, and three antitumor drugs, carboplatin, gemcitabine, and vinorelbine, in two canine mammary breast cancer cell lines. Our results indicate that the curve fitting process necessary for building the synergy reference tridimensional models (Loewe, Bliss or HSA), is the primary factor that causes differences between the two software programs, thereby influencing the final synergy/antagonism scores produced by each software.

## 2. Results

### 2.1. Initial Characterization of Drug Action in Canine Mammary Tumor Cells

**Figure 1:**
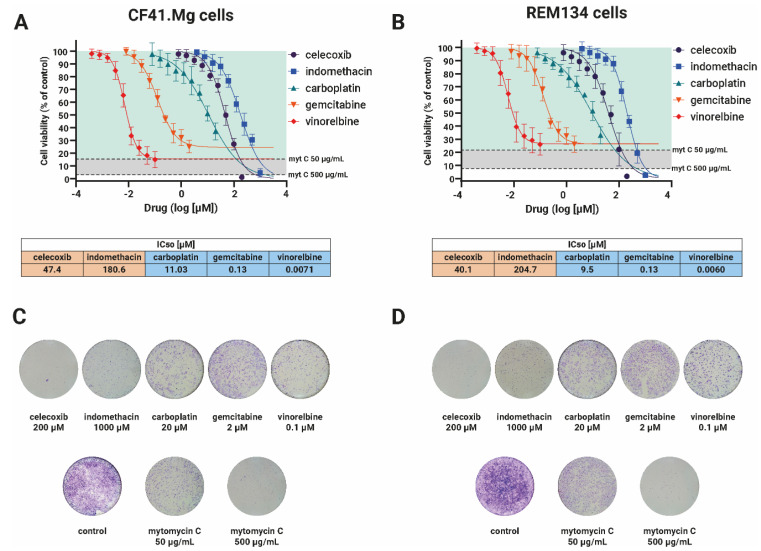
Initial characterization of the drugs used in this study. CF41.Mg and REM134 cells were exposed to two NSAIDs (celecoxib or indomethacin) and three chemotherapy drugs (carboplatin, gemcitabine or vinorelbine). (**A**,**B**) Concentration–response curves for CF41.Mg cells (**A**) or REM134 cells (**B**) were exposed to the drugs. Cells were seeded in 384-well plates and exposed to the drugs for 72 h. Cell viability was measured by resazurin reduction. Cell viability data was loaded in GraphPad Prism and fitted to the four-parameter curve. IC_50_ in the table corresponds to three experiments performed in duplicate. Mitomycin C was used as a positive control of the antiproliferative effect (50 µg/mL) and cytotoxic effect (500 µg/mL). Data points represents the average ± standard deviation from 4 independent experiments. (**C**,**D**) Visual assessment of the cell number after drug exposure. CF41.Mg (**C**) or REM134 cells (**D**) were seeded in 96-well plates and exposed to the drugs for 72 h. Cells were stained with crystal violet stain. The figure shows representatives for 4 independent experiments.

This study first aimed to characterize the five drugs and determine their optimal concentration ranges for further combination studies. The drugs were tested at nine different concentrations using the resazurin reduction method after 72 h of drug exposure. Additionally, another set of cells was exposed to the drugs and stained with crystal violet. Figure 1 presents the results of both experiments, which included the use of mitomycin C as a control at two different concentrations: 50 µg/mL, which have antiproliferative effect [16], and 500 µg/mL, to ensure cytotoxic effect.

Figure 1A,B show that the drugs share a similar behavior between broth cell lines. The EC_50_ for each drug is shown in Figure 1. Among the NSAIDs, celecoxib has a more potent effect than indomethacin in both cell lines, whereas vinorelbine is the most potent antitumor drug, followed by gemcitabine and carboplatin.

It is worth noting that the initial characterization of gemcitabine and vinorelbine revealed that these drugs achieve their maximum effect with nearly 20% of cells remaining, similar to the effect achieved by mitomycin at a concentration of 50 µg/mL. This finding suggests that these drugs act as antiproliferative, which is consistent with what has been reported in the literature [17,18]. In contrast, carboplatin was not able to entirely reduce cell viability at the maximum concentration tested, although the concentration–response curve indicates that the drug could achieve complete cell killing at higher concentrations. However, higher concentrations of carboplatin could not be used due to solubility issues.

We performed a crystal violet stain to confirm the presence of remaining cells at higher concentrations used in the resazurin study. Figure 1C,D illustrate that there were remaining cells at higher concentrations of the antitumor drugs gemcitabine, vinorelbine, and carboplatin. With regard to the NSAIDs, celecoxib at a concentration of 200 µM was able to eliminate all cells, while a few cells remained after exposure to 1000 µM of indomethacin, which is consistent with the cell viability measurements.

### 2.2. Comparison between Software Performance

**Figure 2:**
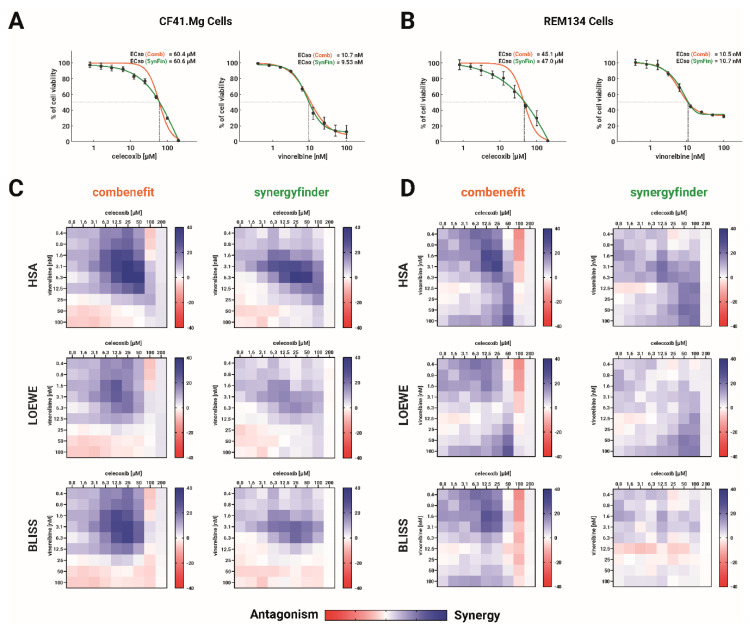
Comparison of curve fitting and combination analysis between software used for drug combinations. CF41.Mg and REM134 cells were seeded in 384-well plates and exposed to 9 different concentrations of celecoxib and vinorelbine, as well as all possible combinations of the two drugs. Cell viability after 72 h was measured by resazurin reduction. The resultant data was then subjected to synergy analysis using Combenefit and SynergyFinder software. (**A**,**B**) The concentration–response curves for CF41.Mg cells (**A**) or REM134 cells (**B**) exposed to celecoxib or vinorelbine were fitted using Combenefit (represented by orange curves) and SynergyFinder (represented by green curves). The EC_50_, which is the concentration of the drug that reduces cell viability by 50% between 100% and 0% in absolute terms, was used to describe the results. Data points represents the average ± standard deviation from four independent experiments. (**C**,**D**) Heatmaps showing the combination outcome over the cell viability when the data was analyzed by Combenefit or SynergyFinder, under the HSA, Loewe and Bliss models for combination analysis. For all heatmaps, blue colors indicate synergistic points, whereas reddish points indicate antagonistic points. N = 4.

Once we established the optimal concentration ranges, we conducted combination studies in a chessboard matrix scheme, as previously described [19]. Six combinations were evaluated: three based on celecoxib (celecoxib-carboplatin, celecoxib-gemcitabine, and celecoxib–vinorelbine) and three based on indomethacin (indomethacin-carboplatin, indomethacin-gemcitabine, and indomethacin-vinorelbine). For each combination, we utilized nine concentrations of each drug, and the viability data was loaded into the Combenefit and SynergyFinder software for analysis. We initially analyzed each concentration–response curve generated by each software. Figure 2 compares the curves generated by each software for the celecoxib–vinorelbine drug pair in both cell lines. A complete analysis for every curve for all drug pairs in CF41.Mg and REM134 cell lines is presented in Supplementary Figures S1 and S2, respectively.

The curve fitting between both software for celecoxib data differed notably, as shown in Figure 2. Vinorelbine curve fitting, however, is similar in both analyses. Combenefit did not adequately fit the curve to the data for celecoxib in CF41.Mg cells, although the EC_50_ for celecoxib was similar between both software. Below the EC_50_, Combenefit tended to fit the curve above the actual drug effect points, and above the EC_50_, Combenefit fit the curve under the actual effect points. A similar trend was noted in REM134 cells and almost every curve generated by celecoxib in this study (Supplementary Figures S1 and S2). The odd curve generated by Combenefit for celecoxib is due to this software forcing the curve fitting between 0% and 100% of the effect (cell viability). Conversely, SynergyFinder does not force the curve, generating a theoretical “maximum effect” under 0% of viability. Although this is an unreal effect, it allows the curve to fit appropriately to the actual data points of this drug.

Both software generated similar curves for drugs whose maximum effect reached a plateau over 0% of cell viability, such as the case of vinorelbine (Figure 2) or gemcitabine (Supplementary Figures S1 and S2).

Each software used the concentration–response curves to build a tridimensional map of effect under the three main models used for drug combinations (Loewe, Bliss, or HSA), after which the cell viability data was compared point by point. Usually, the differences between the model and the actual cell viability data are shown as a heatmap. Figure 2 shows the generated heatmaps for the drug pair celecoxib–vinorelbine, under the Loewe, Bliss and HSA model, by the two software used.

Combenefit-generated heatmaps showed strong synergy spots in the range of 1.6–50 µM of celecoxib in both cell lines. In this range of concentration, curve fitting (and consequently the built model) are over the actual viability data. Thus, viability data is immediately considered synergistic. Because SynergyFinder fits in just the data points, the synergy signal in this range is weaker. On the other hand, the 100 µM celecoxib data is interpreted as antagonistic by Combenefit, because the actual viability is over the model built. That can be seen in the Combenefit heatmaps as a strong reddish line at this celecoxib concentration.

The heatmaps generated by Combenefit revealed pronounced synergy signals in both cell lines within the concentration range of 1.6–50 µM of celecoxib. In this concentration range, the model built by Combenefit exhibited curve fitting that was higher than the actual viability data, leading to instant consideration of the data as synergistic. In contrast, the SynergyFinder software produced weaker synergy signals in this concentration range since it fits in the data points. Conversely, Combenefit identified the data at 100 µM celecoxib as antagonistic because the actual viability exceeds the built model. A strong reddish line indicated this on the Combenefit heatmaps at this celecoxib concentration in both cell lines.

### 2.3. Comparison of Synergy Average between Software

**Figure 3:**
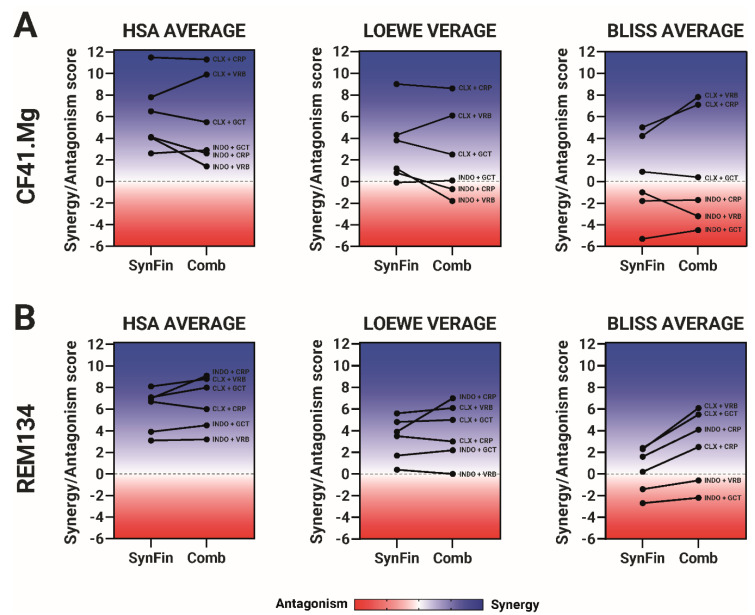
Comparison of the synergy/antagonism averages between the software used for drug combinations. The dots displayed for each software, SynergyFinder (SynFin) and Combenefit (Comb), under the HSA, Loewe, and Bliss models, correspond to the average among each combination point between the drugs tested in a chessboard matrix scheme. The data were obtained from four independent experiments, and raw data given for each software was used to calculate the averages. Blue colors and positive numbers indicate synergistic effects, while red colors and negative scores represent antagonism. (**A**) Averages scores in CF41.Mg cells. (**B**) Averages scores in REM134 cells. Blue colors and positive numbers correspond to synergistic effects, whereas the red zone and negative scores mean antagonism. Graphs summarize four independent experiments.

To enable a comprehensive comparison of the entire combination matrix in both software, we computed the average of each combination under the three studied models, resulting in a general score. The comparison of these scores is visually presented in Figure 3.

As anticipated, the HSA model was the least restrictive when determining synergy, as it assigned synergy scores to all combinations in both software. The Loewe model categorized the INDO+CRP and INDO+VRB combinations in CF41.Mg cells as antagonistic, but only when using Combenefit, while the Bliss model was more stringent in identifying synergy. Consequently, all indomethacin-based combinations were antagonistic in CF41.Mg cells, and the combinations of vinorelbine and gemcitabine with indomethacin were antagonistic in REM134 cells, in both software. All combinations with celecoxib were synergistic under all models, with the exception of the drug pair CLX+GCT, which was scored as additive (not synergistic or antagonistic) in CF41.Mg cells.

It is noteworthy that certain combinations exhibited a significant shift in the strength of synergy, and in some cases, a change in the classification from synergistic to antagonistic, depending on the software used. In CF41.Mg cells, the drug pairs CLX+CRP and CLX+VRB showed greater synergy when analyzed by Combenefit, under the Bliss model. This could be explained by the difference between the actual viability data that is below the fitted curve in the concentration range of 0.8 and 50 µM (Supplementary Figure S1). A similar trend can be seen in CLX+CRP and CLX+VRB combinations in REM134 cells.

On the contrary, in REM134 cells, the synergy score of the indomethacin and carboplatin combination significantly increased when analyzed using Combenefit. The reason for this significant shift can be attributed to the curve fitting of carboplatin by Combenefit. At carboplatin 10 and 20 µM, Combenefit fitted the curve above the actual viability data (Supplementary Figure S2). As the Loewe model assigns greater importance to data at higher concentrations, this disparity between the actual and fitted data leads to a significant increase in the overall synergy score for this drug pair in this particular model.

### 2.4. Comparative Metrics between Software

Differences in the equation used for scoring drug combinations across different software represent a potential source of variability that can confound cross-software comparison. Specifically, while SynergyFinder calculates a combination score by averaging the difference between the actual viability data and the theoretical model [11], Combenefit employs a more intricate algorithm that assigns a value to synergistic points and imposes a penalty on antagonistic points [9]. To facilitate a direct comparison of the synergy scoring between these two software, we opted to use the average score from SynergyFinder and the “sum_syn_ant_weighted” score from Combenefit. In order to standardize the scores, we first generated a simulated data set with fully synergistic or antagonistic effects (0% or 100% viability from the first combination point, respectively) that was equally well fitted by both software, thus eliminating the influence of curve-fitting variability. Next, we compared the combination scores point by point between the two software and found no significant differences. We utilized the HSA, Loewe and Bliss models to generate a combination score matrix from the simulated data, which we then used to normalize the scores obtained from our actual data (Supplementary Figure S3).

**Figure 4:**
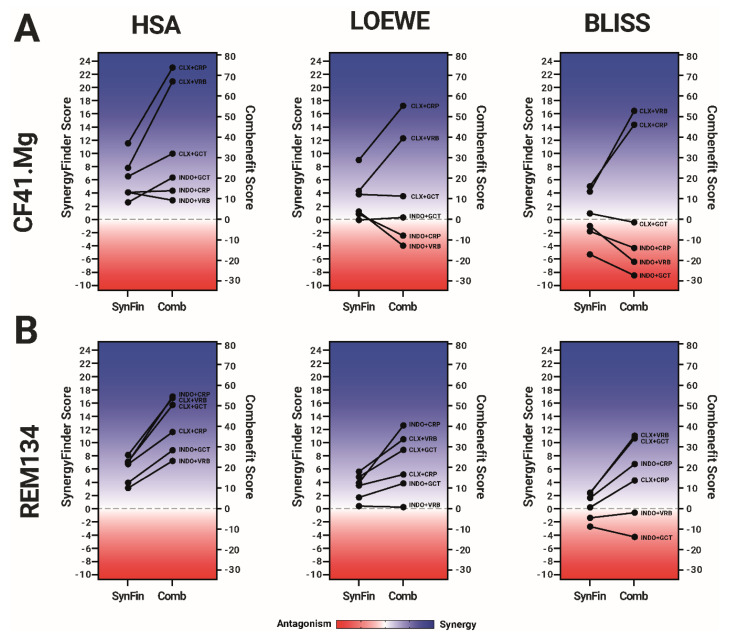
Comparison of the synergy/antagonism metrics between the software used for drug combinations. Dots in the SynergyFinder (SynFin) columns correspond to the average among each combination point between the drugs tested in a chessboard matrix scheme. Dots in the Compusyn (Comp) columns correspond to the “sum_syn_ant_weighted” score, given by this software. The averages are displayed for each software under the HSA, Loewe, and Bliss models. The data were obtained from four independent experiments. Blue colors and positive numbers indicate synergistic effects, while red colors and negative scores represent antagonism. (**A**) SynergyFinder and Combenefit scores in CF41.Mg cells. (**B**) SynergyFinder and Combenefit scores in REM134 cells. Blue colors, and positive numbers correspond to synergistic effects, whereas the red zone, and negative scores, mean antagonism. Graphs summarize four independent experiments.

The normalized scores obtained from both software programs, compared under the three combination models, are depicted in Figure 4. It is worth noting that Combenefit scores exhibit a greater separation between synergistic and antagonistic combinations, yet without affecting the overall pattern of the combinations. Specifically, the major differences are observed in the synergistic combinations rather than in the antagonistic ones.

## 3. Discussion

The ability to accurately predict the synergistic or antagonistic effects of drug combinations is critical for the development of effective cancer therapies. However, achieving this goal can be challenging due to the complexity of the underlying interactions between drugs and cells. In this study, we compared the performance of two widely used software programs, SynergyFinder and Combenefit, in predicting drug combination effects and found that variability in curve fitting was a major factor contributing to differences in their results.

Previous studies comparing the use of SynergyFinder and Combenefit for drug combinations are scarce. For instance, a study evaluated the efficacy of a combination of 5-FU and diosmetin in two colon cancer cells lines using both software. Heatmaps generated from the Highest Single Agent (HSA) analysis revealed inconsistencies in the performance of the two methods in one of the cell lines. While HCT116 cells exhibited similar behavior under both software programs, HT29 cells showed predominantly additive results in Combenefit and antagonistic behavior in SynergyFinder. These findings align with our data, as SynergyFinder consistently demonstrates less pronounced synergistic effects across all models, likely stemming from differences in curve fitting approaches. However, the 5-FU/diosmetin study did not provide further explanations for these disparities [20].

Meyer et al. (2020) conducted a comprehensive review of the drug combination software landscape, including a comparison between SynergyFinder and Combenefit using a large chemotherapy drug dataset. The study found significant inconsistencies when analyzing antimalarial drug datasets using the two software programs, with more conflicting drug pairs under the Bliss model followed by the HSA model. The authors suggested that curve fitting is a variable affecting score differences. The study also showed that fitting the 4-parameter versus the 2-parameter Hill equation could lead to differences in drug effects, such as paclitaxel over RKO cells or azithromycin over *Plasmodium falciparum* strains, similarly to our findings regarding celecoxib [15]. However, while Meyer et al. identified inconsistencies in curve fitting for drugs that failed to achieve complete cell death, our findings reveal a significant difference in a drug that kills all cells but has a curve model that does not conform to the 4-parameter Hill equation (celecoxib in Supplementary Figures S1 and S2). Therefore, because Combenefit utilizes the 4-parameter equation and constrains the curve to fit within the 100–0% range, the resulting curves may have values above or below the actual data points. This phenomenon is confirmed by our simulated data approach, in which data that conforms to the 100–0% effect range produces similar curves between the two software, and only slight variations in the HSA, Loewe, or Bliss models are generated by this data (Supplementary Figure S3).

Many studies employing reference models for drug combinations, such as HSA, Loewe, or Bliss, present only one of them without justification for the selection. Only a handful of studies use all three models to enable comparative analyses. For instance, one study examining new packages for drug combination analysis observed that HSA analysis exhibited considerably more synergy than the Loewe model in a test dataset [21]. Similarly, another study using the fatty-acid hydrolase inhibitor URB597 combined with paclitaxel against mammary and ovarian tumor cells demonstrated that HSA produced higher synergy values compared to Bliss or Loewe models [22]. Even in other models such as *Caenorhabditis elegans*, a similar pattern of consistent changes in synergy trends, as in our study, is observed when comparing the HSA, Loewe, or Bliss models [23].

Our study highlights the importance of presenting complete reports, including original dose–response curves for synergy claims in in vitro studies. Choosing only one model for presenting combination data could be arbitrary without further explanation. Furthermore, reports presenting only HSA-analyzed data warrant special attention, as this model consistently yields synergistic scores. In line with the most up-to-date guidelines for drug combination studies [24].

This study concludes that concentration–response (dose–response) fitting is the main factor that biases the categorization (synergistic or antagonistic) that each software gives to a drug combination. On the other hand, the scores provided by both software are not equivalent, since normalization of them indicates that Combenefit tends to magnify the differences between synergistic and antagonistic combinations. Therefore, we strongly advise reporting complete combination data and the analysis under the three most commonly used reference models for drug combinations.

## 4. Materials and Methods

### 4.1. Cell Lines and Culture Method

We utilized canine mammary tumor (CMT) cell lines CF41.Mg and REM134 in our study. CF41.Mg cells were obtained from ATCC (CRL-6232TM), cultured in high glucose DMEM medium supplemented with 10% fetal bovine serum (HyClone, Pasching, Austria), 2 mM glutamine, and antibiotics (100 U/mL penicillin and 100 μg/mL streptomycin, Biological Industries, Beit HaEmek, Israel), while REM134 cells were obtained from the ECACC (Cat# 12122002) and cultured in MEM medium supplemented with 10% fetal bovine serum (HyClone, Pasching, Austria) and antibiotics (100 U/mL penicillin and 100 μg/mL streptomycin, Biological Industries, Beit HaEmek, Israel). The cells were incubated in a humidified environment at 37 °C with 5% CO_2_. To subculture the cells, we washed them with Phosphate Buffered Saline (PBS) and added 0.5 mL of a solution of 0.25% trypsin and 0.03% EDTA (Sartorius, Beit HaEmek, Israel). After incubating the cells for 1 min, we added 10 mL of complete medium, centrifuged the cells for 8 min at 800× *g*, resuspended the pellet in complete medium, and counted the cells using a Countess II automated cell counter (ThermoFisher Scientific, Waltham, MA, USA). All cell lines were used until passage 20.

### 4.2. Drugs

We employed two non-steroidal analgesics, namely celecoxib (Santa Cruz Biotechnology, Dallas, TX, USA), a selective inhibitor of COX-2, and indomethacin (Cayman Chemical, Ann Arbor, MI, USA), a preferential inhibitor of COX-1. Additionally, we utilized three antitumor drugs, carboplatin (Cayman Chemical, Ann Arbor, MI, USA), gemcitabine (Cayman Chemical, Ann Arbor, MI, USA), and vinorelbine (Santa Cruz Biotechnology, Dallas, TX, USA), that have different modes of action. All drugs were dissolved in dimethyl sulfoxide (DMSO, Merck, Darmstadt, Germany) and assayed at nine different concentrations to generate concentration–response curves and combination models. The DMSO concentration in the cultures never exceeded 0.5% *v*/*v*.

### 4.3. Crystal Violet Staining

Cells were seeded in transparent 96-well plates, 24 h later the drugs were added. After 72 h, the culture medium was removed, and then 100% methanol was added and the plates were left incubating at room temperature for 20 min. The methanol was removed and the plates were washed with distilled water, then a 5% crystal violet (Merck, Darmstadt, Germany) stain was added and left to incubate for 5 min at room temperature. Finally, the plates were washed with distilled water to remove excess stain.

### 4.4. Cell Viability

CMT cells were seeded in black 384-well plates, and after 24 h the drugs were added. After addition of drugs, at 72 h, cell viability was measured by resazurin reduction. Fluorescence was measured on a TECAN Infinity MPRO 200 plate reader (Tecan Männedorf, Switzerland). For resazurin reduction, the culture media were replaced with fresh serum-free media containing 100 µM resazurin. Cells were incubated for 4 h with resazurin, at 37 °C in the dark, and fluorescence was measured at 560 nm (excitation) and 590 nm (emission), on a TECAN Infinity MPRO 200 multimode plate reader.

### 4.5. In Vitro Combinations

Experiments were performed using 9 concentrations of each drug and all possible combinations between them, in a checkerboard matrix. This type of drug trial design is a comprehensive model for the combined drug study, allowing comparisons in combination of the three most common drug combination models: the Loewe additivity model, the Bliss independence model and the HSA model [5]. Cell seeding, base 2 dilutions of drugs and viability measurements were carried out in 384-well plates on an automated robotic system (Opentrons OT-2). The concentration ranges for each drug were indomethacin 3.7–1000 µM, celecoxib 0.7–200 µM, vinorelbine 0.004–0.1 µM, gemcitabine 0.01–2 µM and carboplatin 0.07–20 µM.

### 4.6. Data Analysis

Initial characterizations of the concentration–response behavior of drugs was performed by loading data in GraphPad Prism software (Ver 9.0) and fitting the viability data to the 4-parameter equation. For combination studies, viability data were loaded into the free software Combenefit [9] version 2.02, and SynergyFinder Plus [11]. Both software generated surface response plots by comparing the viability data to a drug combination reference model obtained from the effect of each drug alone. Combenefit utilized t-Student to compare viability data, while SynergyFinder implemented a bootstrapping method. We considered a *p*-value of less than 0.05 to establish statistical differences between drug combinations

## Data Availability

The data presented in this study are available on request from the corresponding author.

## Supplementary Materials

The supporting information can be downloaded at: https://www.mdpi.com/article/10.3390/ijms24119705/s1.
