# Supplementary material for: Mind the Curve: Dose–Response Fitting Biases the Synergy Scores across Software Used for Chemotherapy Combination Studies
Source: Int J Mol Sci. 2023 Jun 3;24(11):9705. doi: 10.3390/ijms24119705 (PMC10253300; doi:10.3390/ijms24119705)
Supplement: Supplementary file 1 [file ijms-24-09705-s001.zip › ijms-2408485-supplementary.pdf]

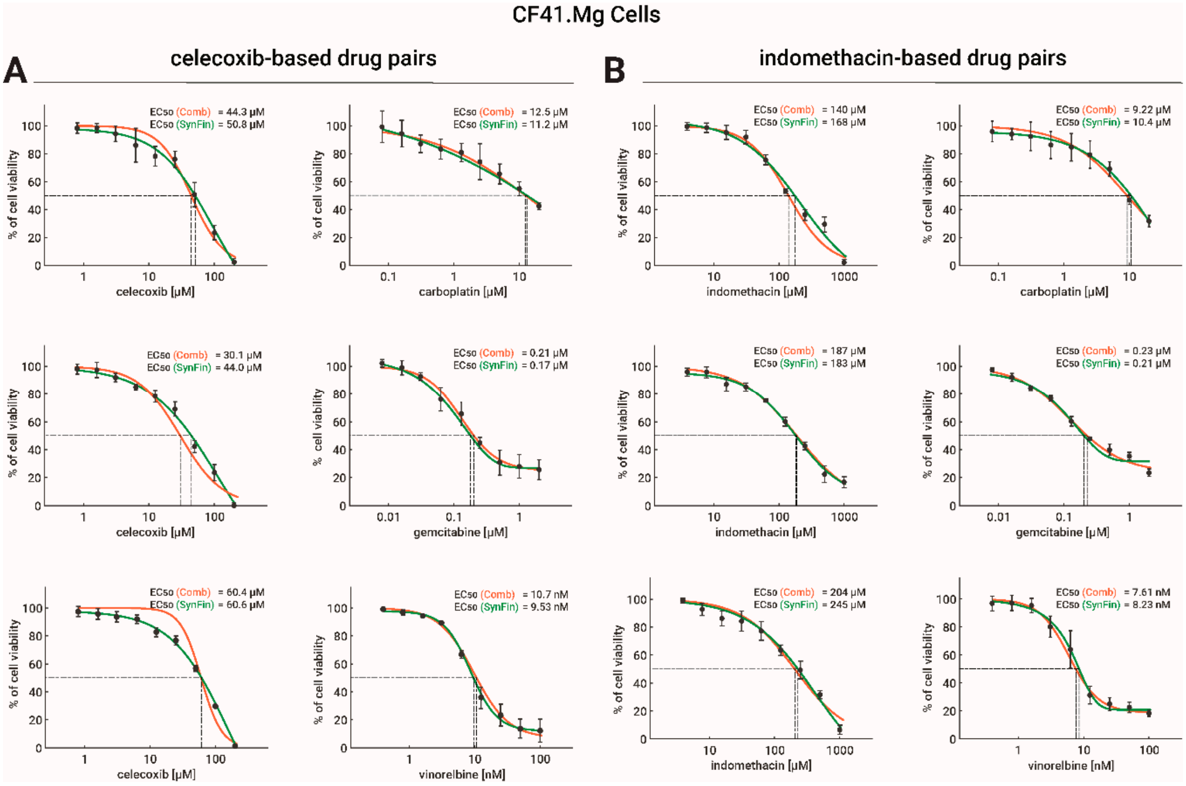

Supplementary Figure S1. Concentration-response curves for the NSAIDs-Antitumor combinations in CF41.Mg cells. CF41.Mg cells were seeded in 384-well plates and exposed to NSAIDs (indomethacin or celecoxib) or antitumor drugs (carboplatin, gemcitabine or vinorelbine) for 72 hours. Cell viability was measured by resazurin reduction. **A.** Celecoxib-based combinations. Curves for celecoxib, carboplatin, gemcitabine and vinorelbine derived from the combinations with celecoxib. **B.** Indomethacin-based combinations. Curves for indomethacin, carboplatin, gemcitabine and vinorelbine derived from the combinations with indomethacin. Data points represents the average ± standard deviation from 4 independent experiments.

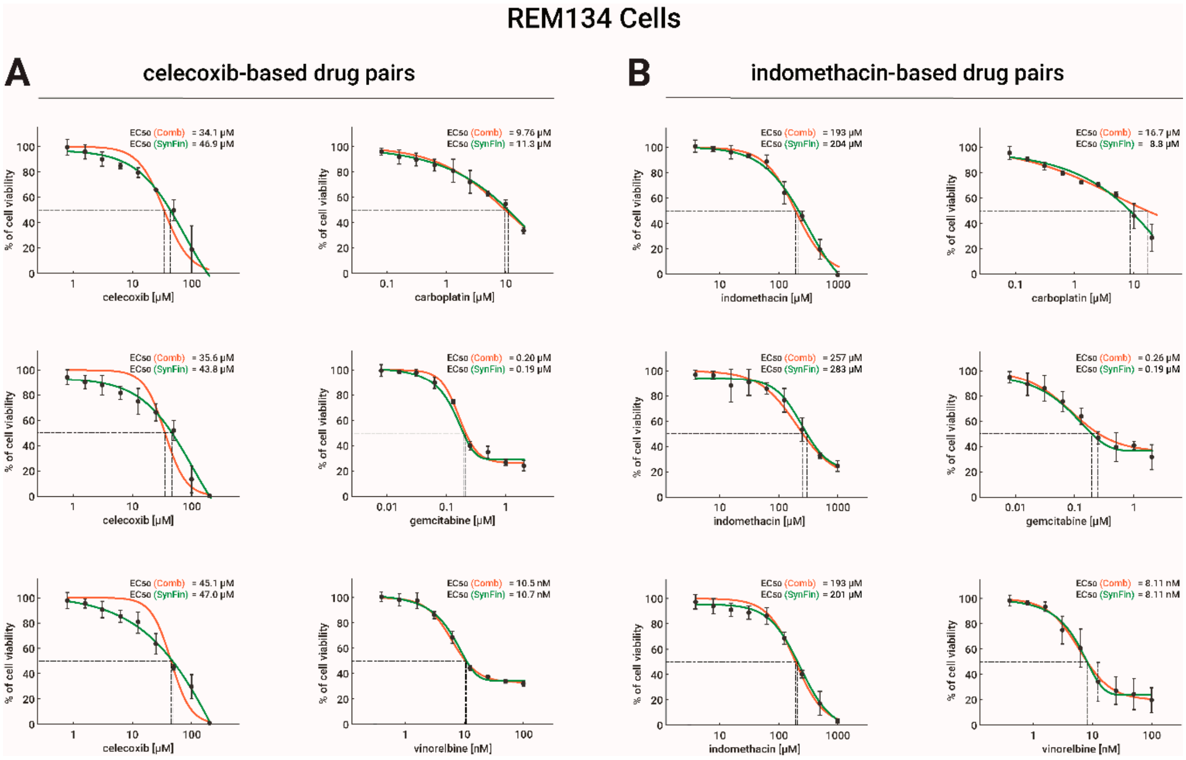

Supplementary Figure S2. Concentration-response curves for the NSAIDs-Antitumor combinations in REM134 cells. REM134 cells were seeded in 384-well plates and exposed to NSAIDs (indomethacin or celecoxib) or antitumor drugs (carboplatin, gemcitabine or vinorelbine) for 72 hours. Cell viability was measured by resazurin reduction. **A.** Celecoxib-based combinations. Curves for celecoxib, carboplatin, gemcitabine and vinorelbine derived from the combinations with celecoxib. **B.** Indomethacin-based combinations. Curves for indomethacin, carboplatin, gemcitabine and vinorelbine derived from the combinations with indomethacin. Data points represents the average ± standard deviation from 4 independent experiments.

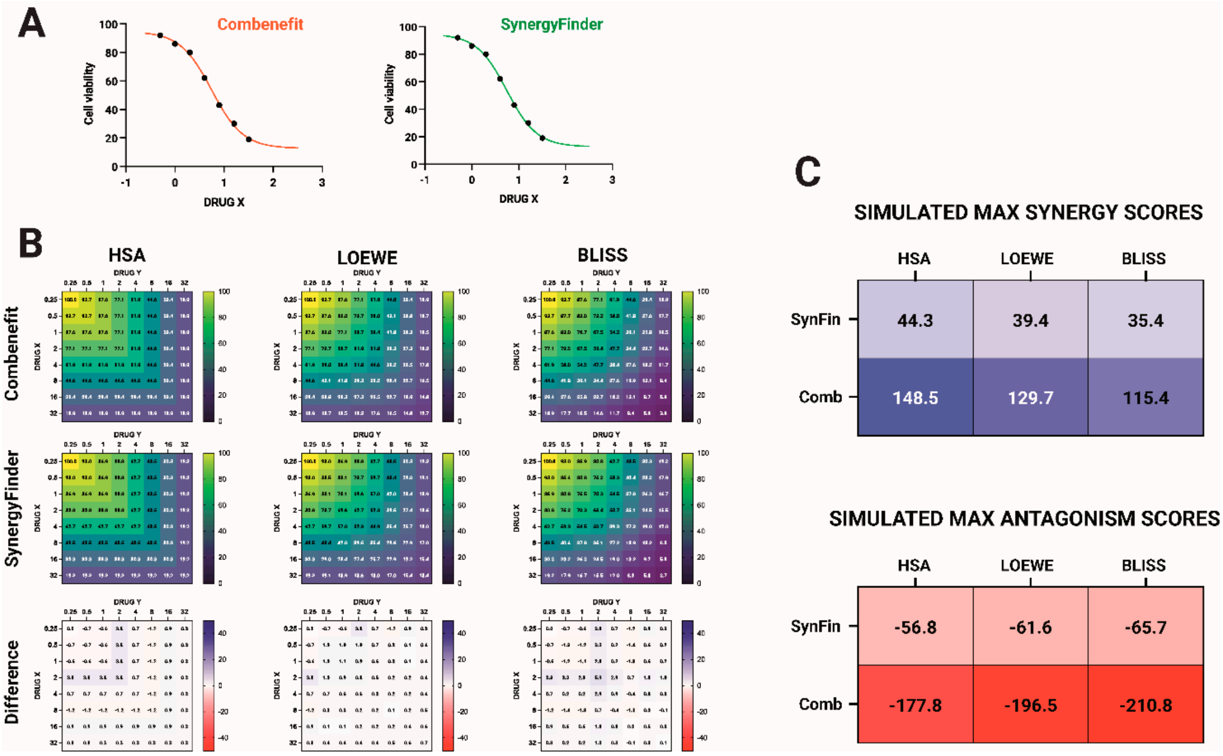

Supplementary Figure S3. Simulated data used to determine the maximum and minimum scores for both synergy and antagonism associated with each software. Simulated data was used to get the same concentration-response curve, and then absolute synergistic or antagonistic data (immediate 0% or 100% viability, respectively) was uploaded to each software. **A.** Simulated data fitted by combenefit and SynergyFinder software. B. HSA; Loewe and Bliss models generated from the simulated data. Viridis color map shows the theoretical viability in each point of the combination if only an additivity effect if seen. Lower row shows the difference between both software simulations. Values of the difference row are expressed as (Combenefit values) – (SynergyFinder values). **C.** Scores for synergy and antagonism got from the simulated data. Positive values represent synergistic combinations and negative values represent antagonistic values.
